# Epidemiology of *Diphyllobothrium nihonkaiense* Diphyllobothriasis, Japan, 2001–2016

**DOI:** 10.3201/eid2408.171454

**Published:** 2018-08

**Authors:** Hiroshi Ikuno, Shinkichi Akao, Hiroshi Yamasaki

**Affiliations:** BML Inc., Kawagoe, Japan (H. Ikuno); National Defense Medical College, Tokorozawa, Japan (S. Akao);; National Institute of Infectious Diseases, Tokyo, Japan (H. Yamasaki)

**Keywords:** *Diphyllobothrium nihonkaiense*, *Dibothriocephalus* *nihonkaiensis*, tapeworm, diphyllobothriasis, cestodiosis, foodborne parasitic disease, epidemiology, Japan, food safety, parasites, raw fish

## Abstract

We report 958 cases of cestodiasis occurring in Japan during 2001–2016. The predominant pathogen was *Diphyllobothrium nihonkaiense* tapeworm (n = 825), which caused 86.1% of all cases. The other cestode species involved were *Taenia* spp. (10.3%), *Diplogonoporus balaenopterae* (3.3%), and *Spirometra* spp. (0.2%). We estimated *D. nihonkaiense* diphyllobothriasis incidence as 52 cases/year. We observed a predominance of cases during March–July, coinciding with the cherry salmon and immature chum salmon fishing season, but cases were present year-round, suggesting that other fish could be involved in transmission to humans. Because of increased salmon trade, increased tourism in Japan, and lack of awareness of the risks associated with eating raw fish, cases of *D. nihonkaiense* diphyllobothriasis are expected to rise. Therefore, information regarding these concerning parasitic infections and warnings of the potential risks associated with these infections must be disseminated to consumers, food producers, restaurant owners, physicians, and travelers.

## Abstract 2

The threat from this disease, the most common cestodiasis in Japan, is increasing because of globalization and lack of awareness.

In Japan, the occurrence of soil-transmitted helminthiases declined sharply in 1949 (*1*). However, foodborne parasitic infections, which are closely associated with the Japanese food custom of eating raw fish, have remained. Diphyllobothriasis caused by the adult tapeworm *Diphyllobothrium nihonkaiense* (proposed as *Dibothriocephalus nihonkaiensis* in 2017) (*2*), an infection closely associated with the consumption of raw Pacific salmon, is the most frequently occurring foodborne parasitic infection in Japan. Paleoparasitologic studies have revealed that diphyllobothriasis has existed in Japan for ≈1,000 years (*3*).

Adult *D. nihonkaiense* tapeworms are ribbon-like and composed of a slender and spatulated scolex (2.4–2.8-mm long and 1.2–1.5-mm wide) with paired slit-like bothria, neck (14.4–16.8-mm long and 1.16–1.28-mm wide), and strobila comprising numerous proglottids (*4*) (Figure 1). The *D. nihonkaiense* tapeworm is parasitic in mammals; brown bear, domestic dog, and humans are their definitive hosts (*5*,*6*). Inside humans, the parasite can grow >10 m in length. Adult worms lay millions of eggs, and these eggs are excreted in feces. The *D. nihonkaiense* tapeworm, as well as other diphyllobothriid species, uses 2 intermediate hosts to complete its life cycle (*4*–*6*). The first intermediate host (species in which the procercoid develops) is probably brackish zooplanktonic copepods (*7*). The first intermediate host is consumed by the second intermediate host, Pacific salmonids, namely cherry salmon (*Oncorhynchus masou*), chum salmon (*O. keta*), and pink salmon (*O. gorbuscha*) (*8*–*10*). In the second intermediate host, procercoids develop into plerocercoids, the larval form needed to infect the definitive host (e.g., humans). *D. nihonkaiense* infections are generally asymptomatic or induce relatively mild symptoms, such as mild diarrhea and abdominal pain (*5*,*6*,*11*).

**Figure 1:**
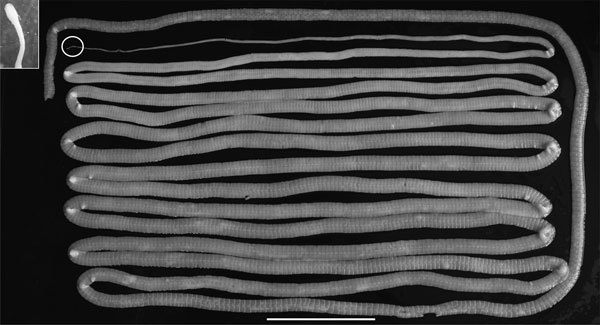
Adult *Diphyllobothrium nihonkaiense* tapeworm expelled from patient, Japan, 2008. Circle indicates the scolex (enlarged in the inset). Scale bar indicates 10 cm.

In Japan, the causative agent of diphyllobothriasis has long been considered to be the tapeworm *Diphyllobothrium latum* (proposed as *Dibothriocephalus latus* in 2017) (*2*), ever since the first case of diphyllobothriasis reported in 1889 (*12*). This belief has caused confusion over diagnostics; whether the cases of diphyllobothriasis reported in Japan in the past were caused by *D. latum* tapeworm or another species was debatable (*13*). However, in 1986, Yamane et al. (*4*) identified the causative agent of diphyllobothriasis as the *D. nihonkaiense* tapeworm from Japan, which is morphologically and ecologically distinct from the *D. latum* tapeworm from Finland. This finding was further verified by DNA analyses (*14*–*16*).

In Japan, all infections, including parasitic infections, linked to the consumption of food should be reported to health authorities as food poisoning, in accordance with the Ordinance for Enforcement of the Food Sanitation Act of 2012. However, despite diphyllobothriasis being the most frequent parasitic infection in Japan, no cases have been duly reported. Thus, diphyllobothriasis epidemiology has been estimated by using only case reports published in journals and the number of outpatients in hospitals (*5*,*17*).

The Department of Bacteriology of BML Inc. (Kawagoe, Saitama, Japan) routinely identifies parasites and diagnoses parasitic infections as requested by physicians from the medical institutions of Japan. During 2001–2016, we examined 632 proglottid samples and 326 egg samples from 958 patients with cestodiasis (Table). In this article, we report the etiologic agents associated with cestodiasis, focusing on diphyllobothriasis, the predominant type of cestodiasis in Japan. We describe the geographic distribution of *D. nihonkaiense* diphyllobothriasis cases and demographic characteristics of patients with this infection. Perspectives of diphyllobothriasis are also discussed.

**Table:** Cestode species identified in patient fecal samples, Japan, 2001–2016

Species	No. samples		Total, no. (%)†
	Proglottid (no.)*	Egg	
*Diphyllobothrium nihonkaiense*	526 (153)	299	825 (86.1)
*Diplogonoporus balaenopterae*	32 (8)	0	32 (3.3)
*Spirometra* spp.	2	0	2 (0.2)
*Taenia* spp.	72 (18)	27	99 (10.3)
Total	632 (179)	326	958 (100)

*Number in parentheses indicates the number examined by molecular analysis during 2012–2016. †Numbers do not add up to 100% because of rounding.

## Identification of Etiologic Agents of Tapeworm Infections

Proglottid and egg samples were collected from patients with diphyllobothriasis and taeniasis in hospitals in Japan. Proglottids were fixed in formalin solution by hospital staff and sent to BML Inc.’s general laboratory for species identification. Almost all proglottids were not attached to a scolex; only 26 (20 diphyllobothriids and 6 taeniids) proglottids had a scolex attached. Fecal samples containing eggs were also collected by hospital staff and sent to BML Inc. These samples were not fixed with formalin; the eggs in the fecal samples were concentrated, and the species were identified on the basis of morphology and egg size. Two *Spirometra* plerocercoids removed surgically from a subcutaneous nodule in the abdomen of 1 patient and a subcutaneous nodule in the ankle of another patient were also received as formalin-fixed samples. We identified proglottids by their morphologic and morphometric markers, such as length and width of mature proglottids and ratio, number, shape, and position of the hermaphrodite genitalia; we also noted the shape and size of the scolex (if available) and the eggs in the uterus (*4*).

During 2012–2016, we identified 179 proglottid samples (161 diphyllobothriids and 18 taeniids) using molecular methods (Table); restriction fragment length polymorphism analysis with PCR-amplified cytochrome *c* oxidase subunit 1 (*cox1*) gene fragment (249-bp long corresponding to base pairs 880–1128) was introduced to confirm diphyllobothriid species (*18*–*20*) and PCR-amplified *cox1* sequencing (145-bp long corresponding to base pairs 641–785) was used for identification of taeniid species (*20*). To amplify the *cox1* gene fragments, we used paired primers 5′-ACAGTGGGTTTAGATGTAAAGACGGC-3′ (forward) and 5′-AGCTACAACAAACCAAGTATCATG-3′ (reverse) for diphyllobothriids (*19*) and 5′-AATTTAGTTCTGCGTTTTTTTGATCC-3′ (forward) and 5′-CTTATWCTRAAACATATATGACTAAT-3′ (reverse) for taeniids (*20*).

**Figure 2:**
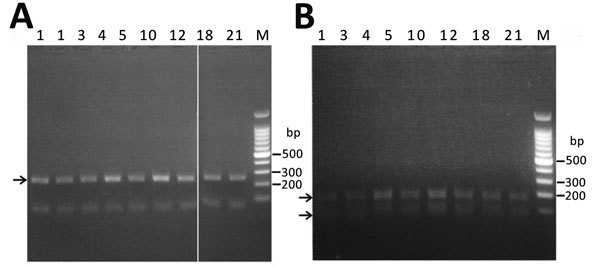
Molecular identification of *Diphyllobothrium nihonkaiense* species by restriction fragment length polymorphism analysis of PCR-amplified *cox1* gene fragments, Japan, 2012–2016. Number above each lane indicates the number of proglottids in the sample. A) Digestion of *cox1* gene fragments (249 bp, arrow) with *Age*I. The leftmost lane is a mock digested sample. *D. nihonkaiense* *cox1* gene did not get cut by the *Age*I enzyme. B) Digestion of *cox1* gene fragments with *BspH*I. The 2 arrows indicate the DNA fragments (164 bp and 85 bp) resulting from the digestion. M, marker.

Of the 958 cestode samples we examined, 825 (526 proglottid and 299 egg, 86.1%) were *D. nihonkaiense*, 32 (3.3%) were *Diplogonoporus balaenopterae* (proposed as *Diphyllobothrium balaenopterae* in 2017) (*2*), 2 (0.2%) were *Spirometra* spp., and 99 (10.3%) were *Taenia* spp. (Table). Of the 179 diphyllobothriid proglottids with which we performed restriction fragment length polymorphism, 153 were confirmed as *D. nihonkaiense* (Figure 2) and 8 as *Dip. balaenopterae*. Of the 18 taeniid proglottids we tested by *cox1* sequencing, 16 were *Taenia saginata*, 1 was *T. solium*, and 1 was *T. asiatica*.

Regarding *Spirometra* plerocercoids, 2 species (*S. erinaceieuropaei* and *S. decipiens*) have been found to be responsible for human sparganosis in Japan (*21*). However, these 2 species were not identified by DNA analysis in this study.

## *D. nihonkaiense* Diphyllobothriasis Annual and Seasonal Occurrence

**Figure 3:**
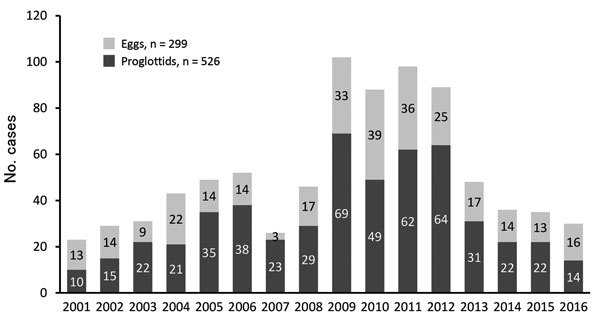
Number of cases of *Diphyllobothrium nihonkaiense* infection, by year, Japan, 2001–2016.

We analyzed the 825 diphyllobothriasis cases attributed to *D. nihonkaiense* infection for their annual and seasonal occurrence. *D. nihonkaiense* diphyllobothriasis occurred persistently, although the frequency varied over the years of the study (Figure 3). Using our data, we estimated that 52 *D. nihonkaiense* diphyllobothriasis cases occurred per year in Japan. The rate of *D. nihonkaiense* diphyllobothriasis cases estimated by examining reports in the literature was ≈40 cases/year (*17*). However, the actual rate is probably much higher and has been estimated to be 100–200 cases/year (*17*).

Although *D. nihonkaiense* diphyllobothriasis occurred throughout the year, the incidence was remarkably higher during March–July, showing a seasonal pattern of occurrence (Technical Appendix Figure 1). Considering that the prepatent period (time from start of infection to time infection is discovered, e.g., person notices strobila excreted in feces) is 2–4 weeks, patients probably acquired infective plerocercoids during February–June. This timing coincides with the season when cherry salmon and immature chum salmon are usually caught and sold. Although specifying the sources of infection is difficult, Pacific salmon are clearly implicated; cherry salmon are caught during March–May, and tokishirazu (i.e., immature chum salmon), which originate from the Amur River in Russia, are caught during May–July (*9*). *D. nihonkaiense* plerocercoids have not been found in akizake (i.e., mature chum salmon), which are not prevalent in fishing waters during February–June because they return to their natal rivers for spawning in autumn (*9*). However, considering that *D. nihonkaiense* diphyllobothriasis occurs throughout the year, akizake and other fish that salmonids eat might also be associated with the occurrence of diphyllobothriasis. Further study is necessary for elucidating this possibility.

## Geographic Distribution of *D. nihonkaiense* Diphyllobothriasis

**Figure 4:**
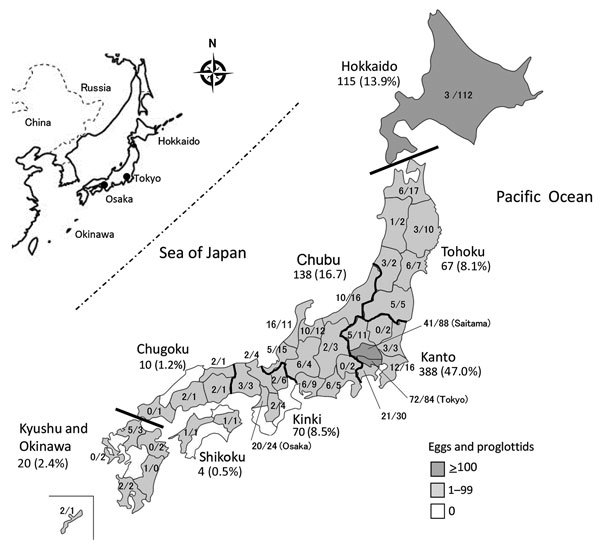
Geographic distribution of patients infected with *Diphyllobothrium nihonkaiense* tapeworm, by administrative region, Japan, 2001–2016. Thick lines indicate divisions between the 8 regions of Japan (Hokkaido, Tohoku, Kanto, Chubu, Kinki, Chugoku, Shikoku, and Kyushu and Okinawa). The total numbers of egg and proglottid samples and percentage of *D. nihonkaiense* infections are given per region. The percentages do not add up to 100% because of rounding. The numbers of egg/proglottid samples are given per prefecture. The prefectures of 13 (1.6%) of 825 patients were unknown; these patients were, therefore, not included. Inset map shows location of Japan in East Asia.

*D. nihonkaiense* diphyllobothriasis occurred widely (40/47 prefectures) throughout Japan, from Hokkaido Prefecture to Okinawa Prefecture. The regions where *D. nihonkaiense* diphyllobothriasis occurred most often were the populous cities of Tokyo and Saitama in the Kanto region (Figure 4), owing to their high consumption of raw Pacific salmon, followed by the Hokkaido Prefecture, Chubu region along the Sea of Japan, Tohoku region (where salmon are caught and consumed locally), and Kinki region (with populous prefectures, e.g., Osaka Prefecture). The incidence of *D. nihonkaiense* diphyllobothriasis was lower in the southern regions than in the northern regions.

From 1979 through the 1990s, diphyllobothriasis occurred mainly in Hokkaido Prefecture, Tohoku region, and along the coastal regions of the Sea of Japan, where salmon are caught and consumed locally (*22*). However, with the rapid advancement of food transportation systems and techniques to retain freshness, diphyllobothriasis spread from the northern parts of Japan to its big cities, such as Tokyo and Osaka, in which salmon consumption has been on the rise since the 1990s.

## Demographic Analysis of Patients and Clinical Signs

During 2012–2015, we conducted a survey to investigate patient demographics. Of the 139 patients who participated, 136 indicated their sex: 85 (61%) were male and 51 (37%) were female. In total, 114 patients indicated their age; age ranged from 2 years to >90 years, but most patients (of either sex) were in the 20–60-year age range (Technical Appendix Figure 2).

Most patients noticed they expelled strobilae when they defecated; for 8 patients, the strobilae were incidentally detected during colonoscopy. Of the 78 patients indicating clinical symptoms, 29 (37.1%) were asymptomatic. Light diarrhea occurred in 28 (34.0%) patients; abdominal pain in 18 (22.0%) patients; abdominal discomfort in 4 (4.9%) patients; and constipation, vomiting, and weight loss in 1 patient each. Most patients experienced mental distress over defecating and discharging proglottids.

## Possible Sources and Locality of Infection

Regarding questions on the consumption of raw fish in the 2012–2015 patient survey, 12 of 15 patients replied that they had eaten dishes containing raw salmon, such as sushi and sashimi. However, the salmon species consumed could not be specified in all cases.

Seven patients had traveled abroad, some to multiple countries: 4 patients went to the United States; 2 patients to South Korea; and 1 patient each to Vietnam, Myanmar, the Netherlands, Belgium, and Italy. However, all 7 patients were considered to have been infected with *D. nihonkaiense* tapeworm in Japan because they had not consumed any kind of raw fish during travel.

The patients with diplogonoporiasis caused by *Dip. balaenopterae* infection were also all infected in Japan. The 17 cases of *T. saginata* and *T. solium* infection that occurred during 2012–2016 were all imported cases, but 1 case of *T. asiatica* infection was acquired in Japan through the consumption of raw pork liver.

## Treatment and Prevention

Praziquantel is recommended as the first-choice anthelminthic drug for diphyllobothriasis (*23*), and this drug was used in all the cases in this study. To prevent recurrence, excretion of the scolex in the feces must be confirmed. If excretion is not detected, further observation of the feces for discharged proglottids or eggs is needed for 2–3 more months.

The most effective prevention method for diphyllobothriasis is to avoid the consumption of raw and undercooked Pacific salmon. If the salmon is cooked at 55°C or frozen at either −8°C for 12 hours or −10°C for 6 hours, plerocercoids in the salmon are killed, and their infectivity is lost (*24*). The US Food and Drug Administration recommends that fish be frozen at −35°C for 15 hours or −20°C for 7 days before consumption of raw or poorly cooked fish (*25*). However, this standard is difficult to achieve in Japan, considering the preference for and, thus, high consumption of traditional raw fish dishes.

In Japan, deep freezing has become a legal obligation to prevent infection with *Kudoa septempunctata* myxozoan parasite in flounder and *Sarcocystis fayeri* protozoon parasite in horse meat. However, this practice has not been implemented for the fishborne parasites *Diphyllobothrium* spp., *Dibothriocephalus* spp., and *Anisakis* spp.

## Perspectives of Diphyllobothriasis

The number of diphyllobothriasis cases attributable to *D. nihonkaiense* infection is expected to rise in Japan, considering this pathogen’s association with Japanese food customs. Also on the rise in Japan is tourism; in 2016, ≈24 million international travelers came to Japan, a 21.8% increase from the year before (http://www.jnto.go.jp/jpn/statistics/visitor_trends/index.html). With the increase in numbers of persons traveling to Japan for sightseeing and business purposes, international travelers acquiring infections with *D. nihonkaiense* tapeworm via the consumption of Japanese foods made with raw salmon, such as sushi and sashimi, is of great concern. In fact, 1 case was reported in a visitor from China (*26*).

Infection with *D. nihonkaiense* tapeworm is no longer a public health problem limited to East Asia and the North Pacific coast of North America; this pathogen is spreading due to the globalization of trade and increased commerce with salmon. Several cases of infection with *D. nihonkaiense* tapeworm have been reported in Europe (*27*) and New Zealand (*28*), where this pathogen was previously absent. The sources of infection for these cases are suspected to be the salmon imported from North America (*27*). Furthermore, regardless of immunity, anyone can get infected with *D. nihonkaiense* tapeworm in the countries where the pathogen exists, such as Korea (*29*,*30*), China (*26*,*31*), the United States (*32*), Canada (*33*,*34*), and eastern Russia (*35*).

Besides infections with *D. nihonkaiense* tapeworm, the following rare and autochthonous cestodes have been sporadically reported in humans in Japan: *Diphyllobothrium stemmacephalum* (*36*), *Adenocephalus pacificus* (*37*), *Dip. balaenopterae* (*38*), and *Spirometra* spp. (*39*). In contrast, human taeniasis has been exclusively reported as imported cases, but *T. asiatica* infections in Japan have been confirmed to be autochthonous infections through the consumption of raw pork liver (*17*,*40*).

From the public health point of view, most of the population in Japan are still unaware of the risk for *D. nihonkaiense* infection associated with the consumption of raw salmon or the risks for infections with other cestodes. Therefore, information regarding parasitic infections and warnings of the potential risks associated with these infections must be disseminated to consumers, food producers, restaurant owners, physicians, and visitors.

## Conclusions

*D. nihonkaiense* diphyllobothriasis is no longer a public health issue limited to only East Asia, including Japan, and North America but is becoming a global threat due to the increasing consumption of raw salmon worldwide. Since 2005, *D. nihonkaiense* diphyllobothriasis has been reported in Europe and New Zealand, where the disease has no endemic foci. Considering these global occurrences, anyone consuming salmon is at risk for *D. nihonkaiense* diphyllobothriasis, not only in Japan and along the North Pacific coast of North America, where local salmon is consumed, but also in Europe, where imported salmon is consumed. The effects of globalization, such as the expansion of the salmon market, the increase in travel to and from diphyllobothriasis-endemic countries, and the global change in eating habits, might cause an increase in the incidence of *D. nihonkaiense* infections worldwide, in places where diphyllobothriasis was previously present and in places where it was not.

Technical Appendix Average number of patients with *Diphyllobothrium nihonkaiense* tapeworm per month and patient sex and age distribution, Japan, 2001–2016.
