# Supplementary material for: Epidemiology of Diphyllobothrium nihonkaiense Diphyllobothriasis, Japan, 2001–2016
Source: Emerg Infect Dis. 2018 Aug;24(8):1428–34. doi: 10.3201/eid2408.171454 (PMC6056094; doi:10.3201/eid2408.171454)
Supplement: Technical Appendix — Average number of patients with Diphyllobothrium nihonkaiense tapeworm per month and patient sex and age distribution, Japan, 2001–2016. [file 17-1454-Techapp-s1.pdf]

Article DOI: https://doi.org/10.3201/eid2408.171454

Epidemiology of *Diphyllobothrium nihonkaiense* Diphyllobothriasis, Japan, 2001–2016

Technical Appendix

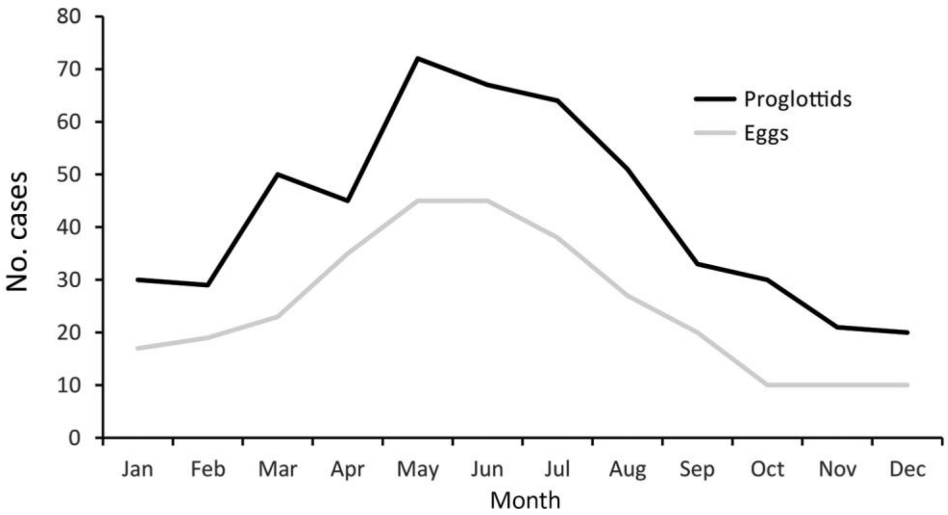

Technical Appendix Figure 1. Average monthly occurrence of *Diphyllobothrium nihonkaiense* tapeworm, by type of sample acquired from patient, Japan, 2001–2016.

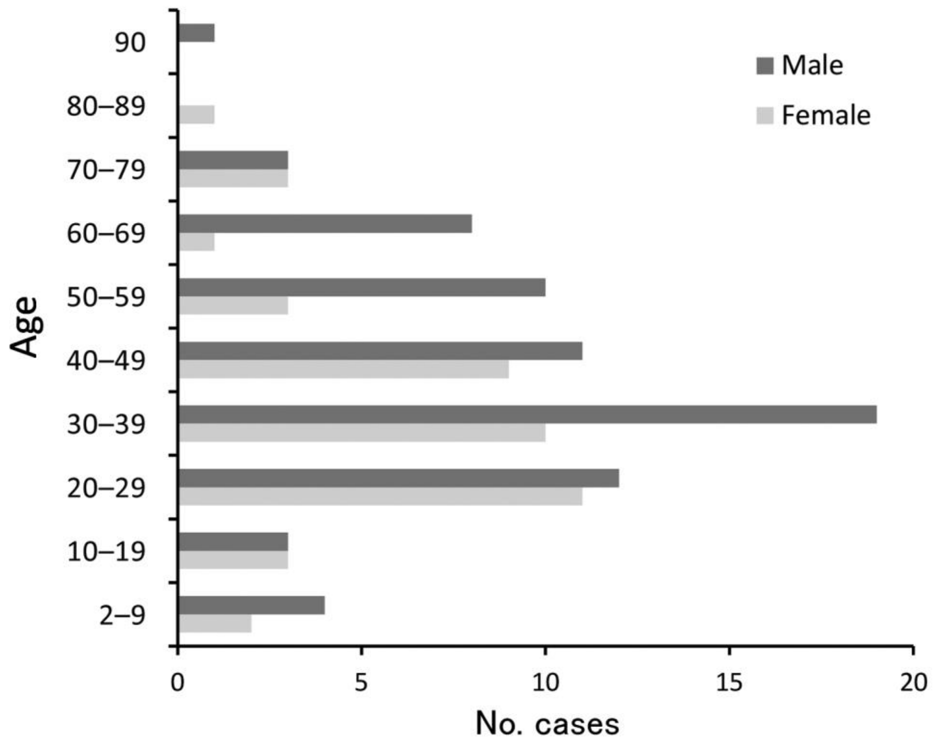

Technical Appendix Figure 2. Age and sex distribution of patients infected with *Diphyllobothrium nihonkaiense* tapeworm, Japan, 2001–2016.
